# Topological Optimization of Circular SAW Resonators: Overcoming the Discreteness Effects

**DOI:** 10.3390/s22031172

**Published:** 2022-02-03

**Authors:** Sergey Yu. Shevchenko, Denis A. Mikhailenko

**Affiliations:** Department of Laser Measuring and Navigation Systems, Faculty of Information Measurement and Biotechnical Systems, Saint Petersburg Electrotechnical University (LETI), Popova Str., h. 5, 197376 Saint Petersburg, Russia; kratosloaded@mail.ru

**Keywords:** surface acoustic wave, SAW resonator, interdigital transducer, ring-shaped design, FEM, lithium niobate, SAW sensor, acceleration measurements

## Abstract

Recently, we proposed a ring-shaped surface acoustic wave (SAW) resonator sensitive element design, as well as analyzed its characteristics and suggested its optimization strategy, with major focus on their temperature stability. Here, we focus on further optimization of the design to narrow the bandwidth and improve signal detection, while taking into account typical technological limitations. Additionally, the purpose of design optimization and modeling is to check the preservation of operability in the case of lithography defects, which is the most common technological error. For that, we suggest structural alteration of the interdigital transducer (IDT) that leads to its partial fragmentation. Using COMSOL Multiphysics computer simulations, we validate several IDT options and show explicitly how it could be optimized by changing its pin geometry. Based on the results of the study, prototyping and printing of ring resonators on a substrate using photolithography will be carried out.

## 1. Introduction

Currently, one of the main components of most modern devices can be called a microelectromechanical system (MEMS), which combines minimal dimensions due to the placement of elements on a single board, low cost due to mass production and low energy consumption at the level of units of watts. Additionally, MEMS have a high measurement frequency. Due to the technologies used in the manufacture of microelectronics, the size of the sensors can be reduced to a match head, but the accuracy and mechanical strength, in most cases, will decrease. The last two parameters, considered to be disadvantages, are not decisive in the consumer segment, which allowed MEMS to become widespread in medicine [1], sports [2], the gaming industry [3] and especially in portable technology [4,5,6].

From the late 1980s to the present, accelerometers were also implemented as MEMS, which allowed them to spread in devices such as smartphones [7], gamepads [8], motion controllers [9], hard disks [10], car digital video recorders (DVRs) [11], and many others. The peculiarities of micromechanical accelerometer (MMA) fabrication are explained by a variety of physical effects underlying the sensors, the materials used, and the technological methods. The simplest MMA is performed on silicon wafers using photolithography, isotropic etching, and deposition of metal or resistive films. Advanced microaccelerometers are technologically sophisticated and can perform the integration of a MEMS device and a CMOS computing core (a multi-core microprocessor chip by design—“system-on-a-chip”). It is worth noting that the production of combined-type sensors has been growing recently, which requires an individual approach during production and testing.

Classic microaccelerometers have the same disadvantages as all MEMS sensors—low accuracy and mechanical strength. Low strength is associated with the low strength characteristic of torsion bars, which leads to their inability to withstand overloads caused by excessive acceleration and / or external mechanical forces.

In recent years, more and more attention has been paid to sensors based on surface acoustic waves (SAW), because the characteristics of MEMS-SAW can surpass their analogs in some parameters, and such sensors can be competitive in the world market.

SAW sensors, although less developed today, represent a reasonable and largely promising alternative. SAW sensors in their design do not have torsions, and the sensitive element is rigidly fixed to the sensor body, which allows it to withstand much higher overloads compared to classical MEMS. Recent developments based on monolithic solid structures are characterized by the relatively high stability of parameters and low power consumption (0.5–1 W) [12]. At the same time, the variations in sensor design based on the effect of acoustic waves are almost limitless. So, it is possible to build sensors on surface acoustic waves, sensitive to small constant signals [13], as well as angular motion sensors on bulk acoustic waves [14], under the influence of which the polarization of waves changes.

Currently, SAW accelerometers are created by a small number of companies [15], and SAW sensors are most widely used as systems for steam and gas analysis [16], temperature control [17], and pressure determination [18]. One of the important stages in the design of SAW devices is mathematical modeling, which, using computer programs, makes it possible to calculate the topology of devices and perform a preliminary calculation of their technical characteristics before the stage of creating prototypes. The main research in the field of SAW accelerometers and similar sensors is aimed at finding new piezoelectric materials for the console of sensitive elements (SE), which could overcome the typical limitations of existing materials (SiO_2_, LiNbO_3_).

Our research is aimed at improving the designs of the sensitive element of rectangular and triangular shapes [19], which now have a drawback in the form of one-sided attachment of the console of the piezoelectric element to the sensor body and, as a result, the load is distributed unevenly. Previously, we proposed a SAW-based MMA design based on a ring-shaped sensitive element [20,21] and considered the optimal mounting of the console in the housing, a material for a promising SE design in accordance with its frequency characteristics, and evaluated the potential effect of external influences, such as excessive acceleration and temperature on SE [22]. The previous work also revealed a wide bandwidth, so this paper considers the optimization of the previously proposed design and reducing the bandwidth with a decrease in the sidelobes of the harmonics. The work was carried out using a computer simulation in the COMSOL Multiphysics software package.

## 2. Sensitive Element Design

**Figure 1:**
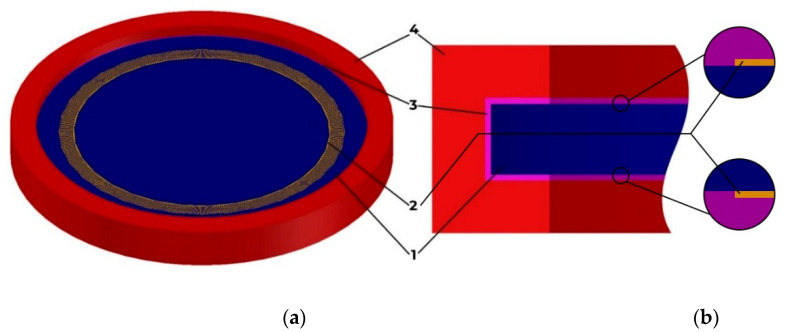
Console attachment methods. General view (**a**) and front view (**b**): 1: console; 2: interdigital transducer; 3: silicone adhesive; 4: housing.

A general view of the sensitive element of the membrane is used from work [22] with fixing the console to the body using silicone adhesive (Figure 1). The model was built in AutoCAD and then imported into COMSOL Multiphysics due to the limited capabilities of the latter’s CAD editor. The resonator consists of two interdigital transducers (2) and a piezoelectric crystal located between the transducers (1). The entire structure is limited both in depth and in radius by a damping medium to suppress parasitic wave reflections from the outer boundaries.

An interdigital transducer is a device that is designed to “convert” electromagnetic waves into SAW and vice versa. The transducer consists of two groups of metal pins (electrodes) nested towards each other and located on the surface of the piezoactive sound conductor.

If an alternating electric voltage is applied between the two poles, then due to the inverse piezoelectric effect, mechanical stresses that periodically change in sign appear almost on the surface of the piezo substrate, leading to the excitation of SAW. When the SAW passes through the IDT structure, an alternating voltage is induced on the electrodes due to the direct piezoelectric effect; that is, the SAW energy is converted back into electrical energy.

The period of the converter must be equal to the wavelength of the coupling with the SAW to be effective—this circumstance determines the frequency of the applied voltage. Due to symmetry, the transducer excites surface acoustic waves in both opposite directions with equal efficiency, i.e., it works bi-directionally. As a rule, a wave propagating in only one direction is used, while the unused wave is absorbed by applying a special coating to the surface, which is a material with high attenuation.

**Figure 2:**
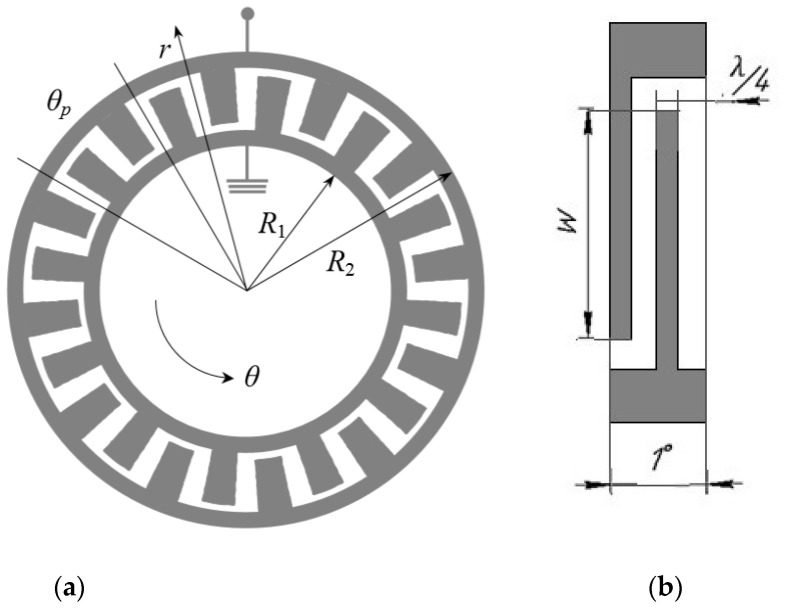
Interdigital transducer [22]. General view (**a**) and construction of one angular period (**b**).

The general scheme of the IDT for this work is shown in Figure 2. The following IDT parameters are used in this work: the length of the IDT period in the center of the ring is 19.2 µm with the angular period of the transducer *θ_p_* = 10 and the height h = 0.2 µm. Taking this value as a wavelength and considering that SAWs decay at a depth of about three wavelengths, the height of the structure will be seven wavelengths, or 134.4 microns. The overhang of the console is 1500 microns. Table 1 shows the overall parameters of the console and IDT.

The characteristics of the materials used are presented in Table 2, Table 3, Table 4 and Table 5. For this work, a lithium niobate material was chosen since it has the highest sensitivity and strength in comparison with other materials presented in [22]. Additionally, a sensitive element with a lithium niobate substrate is easier to manufacture than aluminum nitride, since aluminum nitride is a film material and must be applied to a substrate, for example, quartz.

## 3. Interdigital Transducer Geometry

The properties of the IDT are completely determined by their design characteristics, namely, the geometry of the electrodes (variable width of the electrodes, apodization—a change in the mutual overlap of adjacent electrodes along the length of the IDT according to some functional law) and their location (aperture, periodicity, tilt, number of electrodes), as well as the mutual location of the transducers determining the signal delay time and the relative unevenness of the phase characteristic, the possibility of front correction (for example, with film waveguides) and the branching of SAW energy into adjacent channels. The method of connection to common potential buses (capacitive, optical, multiphase, with potential division) also has a considerable influence.

A simple IDT has a constant spatial period and electrode aperture length and frequency response (sin x)/x with low selectivity. To increase the selective requirements, you can use various methods of weighting (amplitude or apodization) of the IDT, which are achieved by changing, for example, the period, length, and width of the electrodes.

Since the bandwidth of an IDT is inversely proportional to the number of its electrodes, reflections in an equidistant apodized IDT strongly increase, which can be reduced by using an IDT structure with split electrodes, with thinned electrodes, or with electrode breaks.

The transducer is a frequency-selective element; therefore, its amplitude-frequency characteristic has a maximum at the frequency of acoustic synchronism *f*_0_ and is described by the expression

(1) $$H\left(f\right)=N\times A\frac{\sin\left(\pi N\frac{f-f_{0}}{f_{0}}\right)}{\pi N\frac{f-f_{0}}{f_{0}}}.$$

The acoustic synchronism frequency is defined as f0=VΠ2hel, where hel=λΠ2.

The passband is characterized by the number of electrode pairs *N* and is determined by the level 0.707H(f_0_): Δf=1T=VPL=VΠNλP=f0N.

**Figure 3:**
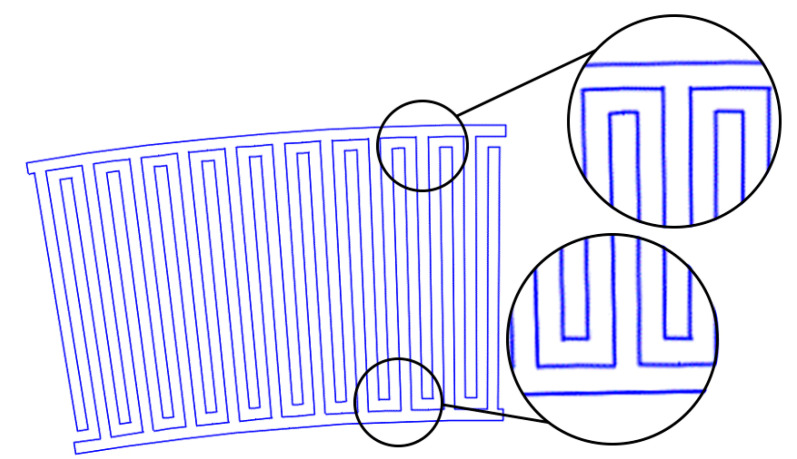
The first type of IDT and initial view of the IDT. All pins are tapered. In all areas from the outer to the inner radii, equal distances (*π*/4) are observed.

**Figure 4:**
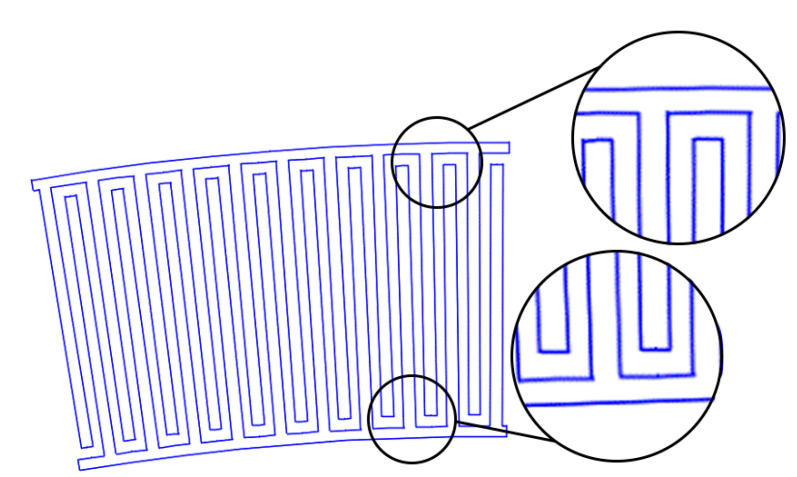
The second type of IDT. The pins are rectangular. At the outer radius, a period of 19.2 μm is observed.

The first part of our work was to determine the most efficient geometry of the interdigital converter and select the shape of the pins:

1. Period constancy: maintaining equal spacing between pins at all distances from the IDT center. In this case, the pins are tapered. (Figure 3);

2. Pins persistence: preservation of the rectangular shape of the pins (Figure 4).

**Figure 5:**
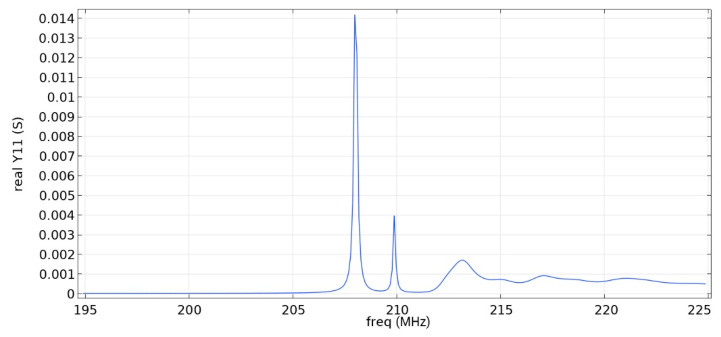
Real component of admittance for the first type of IDT.

**Figure 6:**
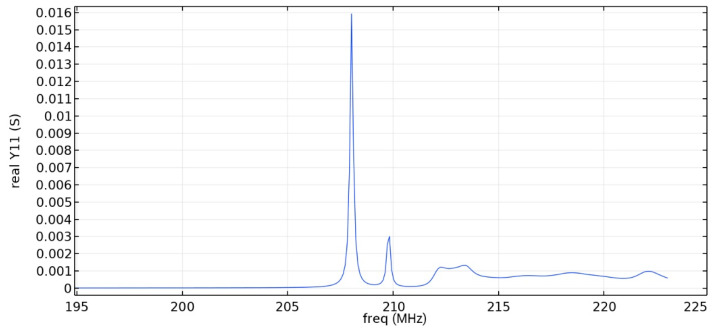
The real component of admittance for the second type of IDT.

Frequency response simulations were performed in COMSOL Multiphysics for a LiNbO_3_ piezoelectric material console in the range 190 MHz to 230 MHz. Figure 5 and Figure 6 and Table 6 show the simulation results for the first and second types of IDTs, respectively.

As can be seen from the simulation results, the constancy of the pins gives better results: the value of the maximum of the first mode (0.01592 S) is more than five times higher than the value of the maximum of the second mode (0.003 S), which corresponds to the three-sigma rule. In addition, the bandwidth is 107 kHz, which is small compared to the data obtained in [22] and with the first type of IDT. You can also notice that Figure 1 differs from the results presented in [22]. This is due to the fact that in this study, the mesh for modeling was changed and applied more densely.

With further optimization of the IDT design, the IDT geometry with pins will be used.

## 4. Selective Pin Removal

The main way to change the IDT in operation is to remove a certain number of electrodes from the structure with a change in the common bus. The absence of a common bus, according to the SAW theory, leads to autogeneration of the wave.

Today, it is technologically difficult to spray a resonator in the form of a ring; therefore, the possibility of transducer segmentation is considered in this work. The quality and method of making prototypes largely determines the characteristics of surfactant devices. The most widespread precision method of applying topology to a piezoelectric substrate is photolithography—a method of creating fragments on the surface due to the sensitivity of coatings to intense energy radiation, so it is possible to recreate a certain mutual arrangement and shape of specified elements [23].

There are three main types of photolithography: contact, projection and maskless laser (electron beam).

Contact photolithography is used for the prototyping and production of small series products. For this type, cheaper and simpler equipment is used than other types of photolithography. During the operation of the device, a special template fits snugly to the semiconductor wafer, and a photoresist is preliminarily applied to its surface. A mercury or LED lamp illuminates the topology image, while its wavelength is responsible for the minimum parameters of the produced fragment located on the plate [23]. Modern precision indicators of contact photolithography equipment are 0.5–1.0 microns. This type has several disadvantages: a limited number of cycles (no more than 70) and a decrease in product quality for each subsequent release.

To reduce low-quality products due to contact, a method of lithography with a micro-gap was developed, the essence of which is that the photographic template is “moved away” from the substrate itself by several microns. This made it possible to process the plate completely in one go. So, this method has become widely used in the serial production of products with an accuracy of about 1 μm [23].

Projection photolithography is used in the manufacture of semiconductor devices, which excludes the use of the contact method, since the minimum parameters of the topological fragment of the equipment (up to 20 nm) are much less than the resolution limit of machines for the contact method of production. The main advantage of the method is the absence of contact between the photomask and the photoresist on the plate. This way, the template is not damaged and can serve for a long time. It is also possible to achieve a minimum resolution of 20 nm.

For maskless laser photolithography, focused laser radiation sources or an electron column generating a focused electron beam are used to illuminate the photoresist and create the desired topology (picture) on a substrate or photomask. A focused laser beam illuminates the topology image, while its wavelength is responsible for the minimum parameters of the produced fragment located on the plate [23].

Contact photolithography equipment is significantly less expensive, making it cost effective for use in R&D labs, universities, research centers, and small-scale production.

The main way to change the IDT in operation is to remove a certain number of electrodes from the structure with a change in the common bus. The absence of a common bus, according to the SAW theory, leads to self-generation of the wave.

A weighing method that does not change the degree of overlap between electrodes of different polarity is called the selective removal method [24]. The principle is to selectively exclude some surfactant sources from the original non-apodized IDT.

Due to additional sampling of the impulse response and interference of waves from different groups of electrodes, harmonic responses appear in the AFC of the converter, the level of which near the passband is 35–40 dB and increases to 15–20 dB with a frequency detuning (by about 10 bands). Weighting by selective removal of electrodes more accurately approximates the given impulse response with an increase in the number of electrodes; hence, the method is suitable for the implementation of narrow bandwidths [23].

As accurate as photolithography is, as with any manufacturing process for parts, there is a potential for defects. Within the framework of this work, one of the tasks is to determine how many unprinted pins and bus sections (which can also be considered forced selective removal of pins) will be an acceptable defect in which the system will be operational.

## 5. Computer Simulation

**Figure 7:**
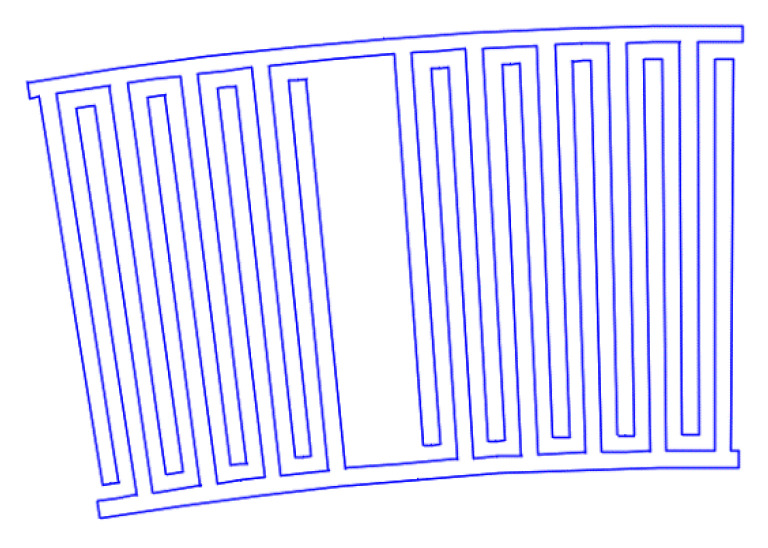
The third type of IDT. Selective withdrawal. Every 10 pair of pins is removed, but there is a common bus.

**Figure 8:**
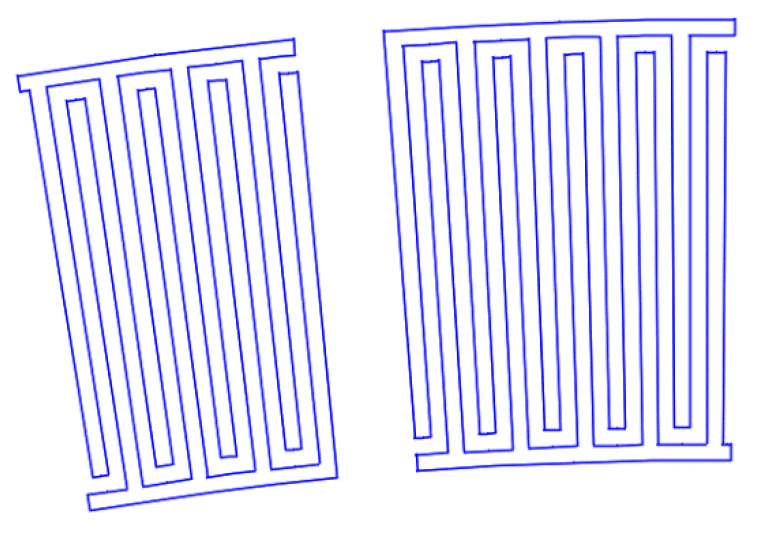
The fourth type of IDT. Selective withdrawal. Every 10 pair of pins is removed. There is no shared bus.

**Figure 9:**
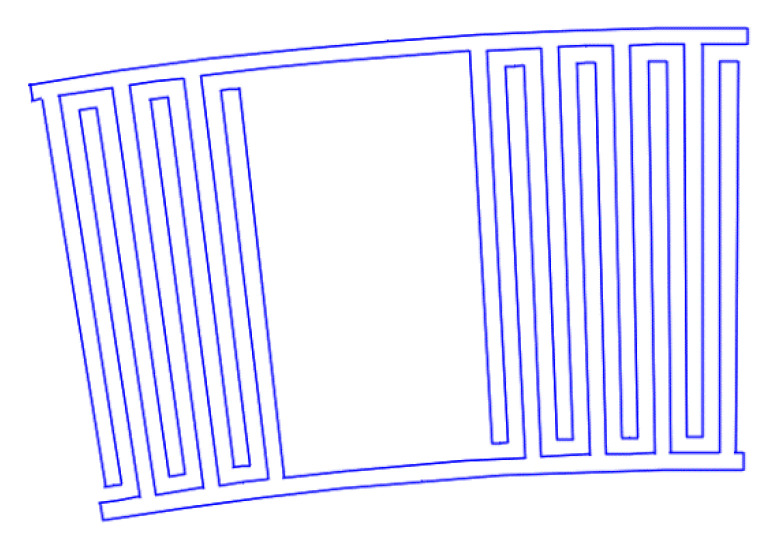
The fifth type of IDT. Selective withdrawal. Three pairs of IDTs are deleted every 10 periods. There is a common bus.

**Figure 10:**
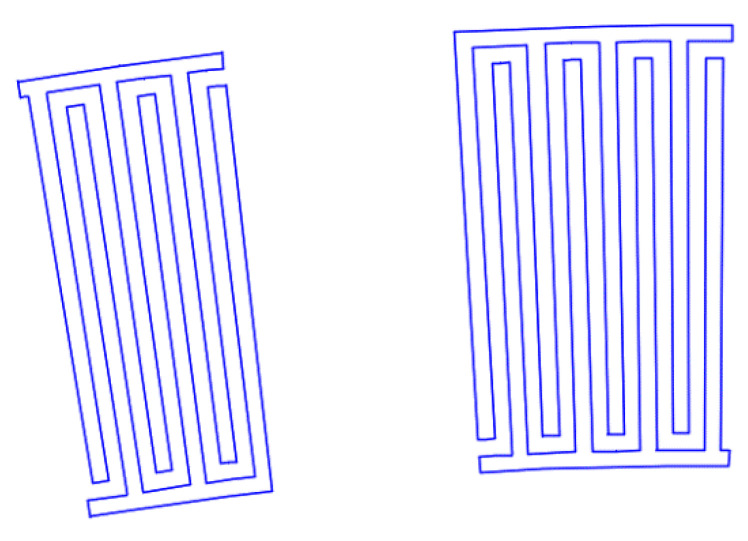
The sixth type of IDT. Selective withdrawal. Three pairs of IDTs are deleted every 10 periods. There is no shared bus.

**Figure 11:**
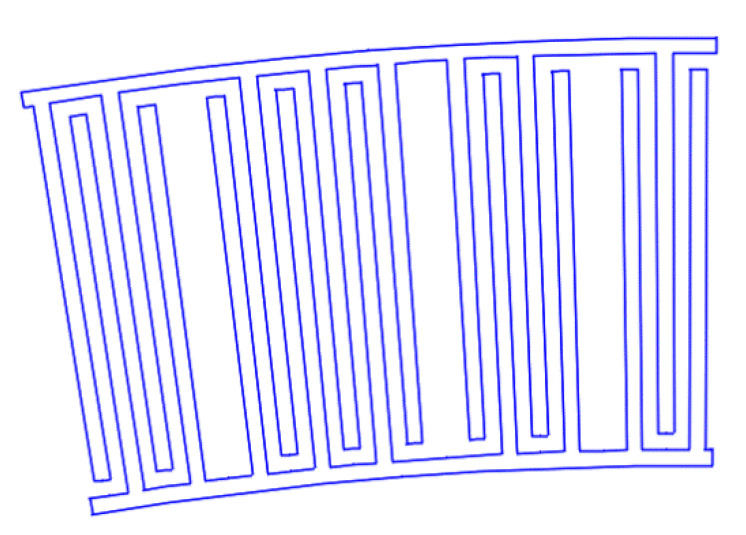
The seventh type of IDT. Selective withdrawal. One of the pins is removed every 3–4 pairs of IDTs. There is a common bus.

**Figure 12:**
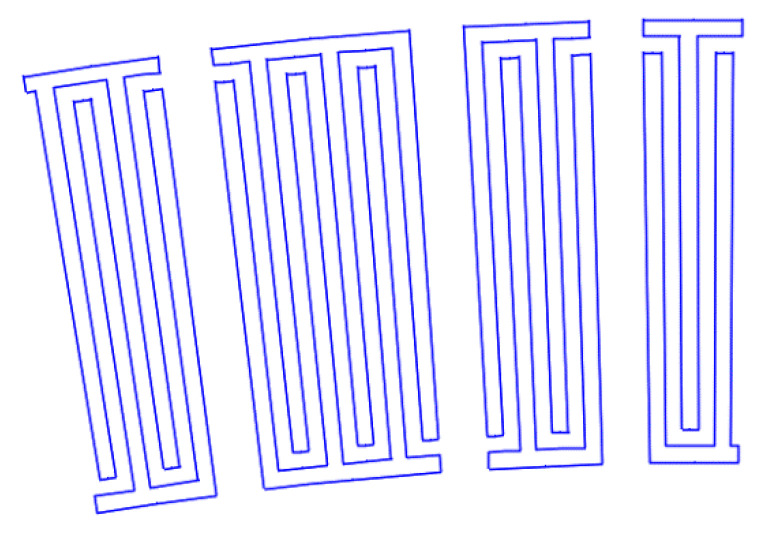
The eighth type of IDT. Selective withdrawal. One of the pins is removed every 3–4 pairs of IDTs. There is no shared bus.

**Figure 13:**
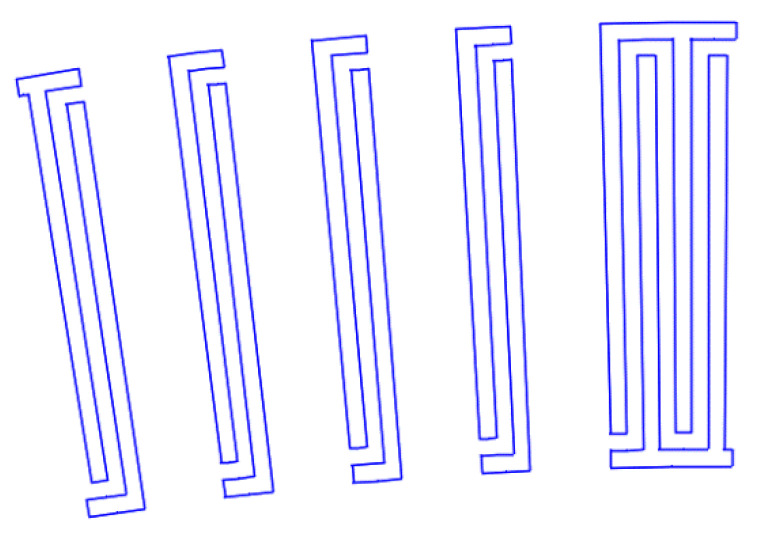
The ninth type of IDT. Selective withdrawal. Every other pair of pins is removed. There is no common bus.

Figure 7, Figure 8, Figure 9, Figure 10, Figure 11, Figure 12 and Figure 13 show the types of IDTs, the modeling of the characteristics of which was carried out in the second part of the work.

As can be seen from Figure 7, Figure 8, Figure 9, Figure 10, Figure 11, Figure 12 and Figure 13, the main method of design modernization is selective IDT retraction.

Frequency response simulations were performed in COMSOL Multiphysics for a LiNbO_3_ piezoelectric material console in the 190 MHz to 230 MHz range. Figure 14, Figure 15, Figure 16, Figure 17, Figure 18, Figure 19 and Figure 20 show the simulation results for all types of IDTs shown in Figure 7, Figure 8, Figure 9, Figure 10, Figure 11, Figure 12 and Figure 13.

As can be seen from the graphs, the resonance frequency is different in all cases and has a spread in the range of 207.9 MHz–211.4 MHz since the surface of the console has a different percentage of metallization.

Analyzing the data obtained, we can say that the selective removal of pins with an interrupted bus is not an optimization of our proposed design. The most effective structure obtained is the third type of IDT: removal of one period with a common bus. The value of the maximum of the first mode (0.00906 S) is three times higher than the value of the maximum of the second mode (0.003 S), which corresponds to the three-sigma rule. The maximum volumetric acoustic wave value is 0.00126 C. The bandwidth is 138 kHz. The bandwidth and the maximum value of the first mode for all types of IDT are presented in Table 7.

The use of an IDT of the fourth type is possible when creating a sensitive element, since the ratio of the maximum values of the first mode to the second is three, and the first mode significantly exceeds the signal from bulk acoustic waves.

The fifth and seventh types of IDTs are not recommended for use, since the maximum values of the first and second mods are similar, which will negatively affect the determination of the output signal at some acceleration values.

The sixth, eighth and ninth types of IDTs are not recommended for use either because of the small useful signal in relation to the signal from the bulk acoustic waves. Due to the improvement in metallization, the conductivity value increased.

When comparing similar types of IDTs (3–4, 5–6, 7–8), we can say that the common bus in the ring resonator allows you to keep the SAW inside the IDT aperture, save more energy and obtain a larger signal.

## 6. Conclusions

The most effective geometry for a SAW ring resonator is rectangular pins, but cone-shaped pins can also be used to create a sensitive element.

Narrowing the periods towards the inner part of the structure improves the frequency characteristics of the ring resonator on surface acoustic waves, namely:

- increase the ratio of the maximum values of the first mode to the second;
- reduce bandwidth.

The preservation of the working capacity of the resonator can be carried out by removing no more than one pair of IDTs for 10 or more periods. In this case, the withdrawal of IDTs should be uniform. With an increase in the number of IDT withdrawals, the geometry of the ring resonator is violated and the wave escapes from the position specified by the geometry.

The presence of a common bus allows you to keep the surface acoustic wave inside the IDT structure.

## Figures and Tables

**Figure 14:**
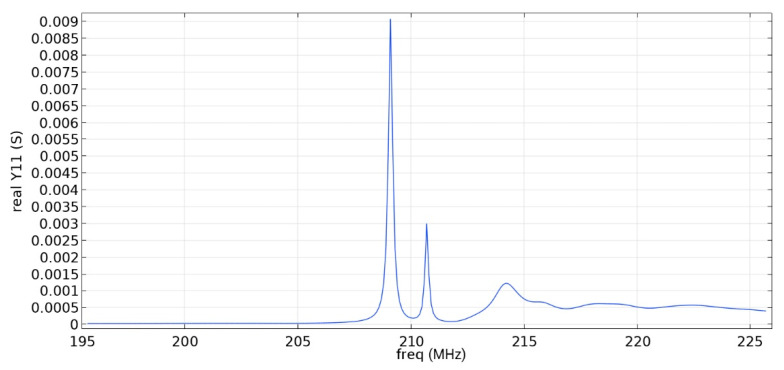
The real component of admittance for the third type of IDT.

**Figure 15:**
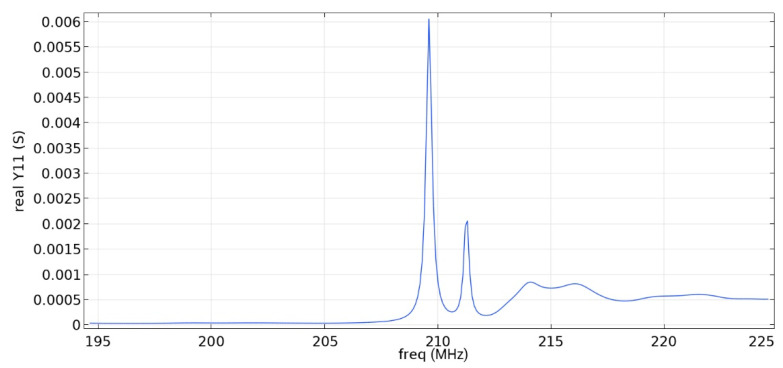
Real component of admittance for the fourth type of IDT.

**Figure 16:**
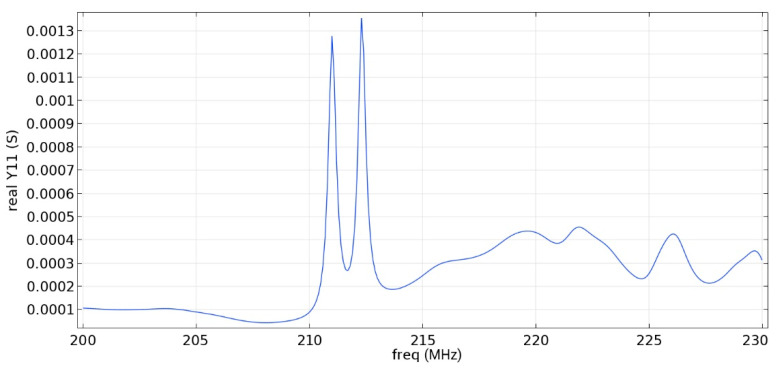
Real component of admittance for the fifth type of IDT.

**Figure 17:**
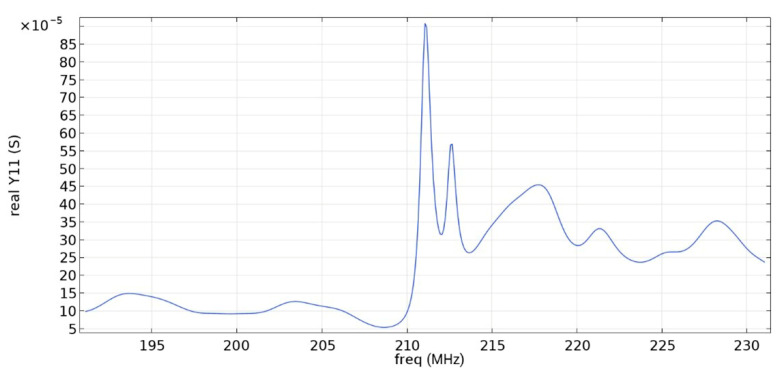
Real component of admittance for the sixth type of IDT.

**Figure 18:**
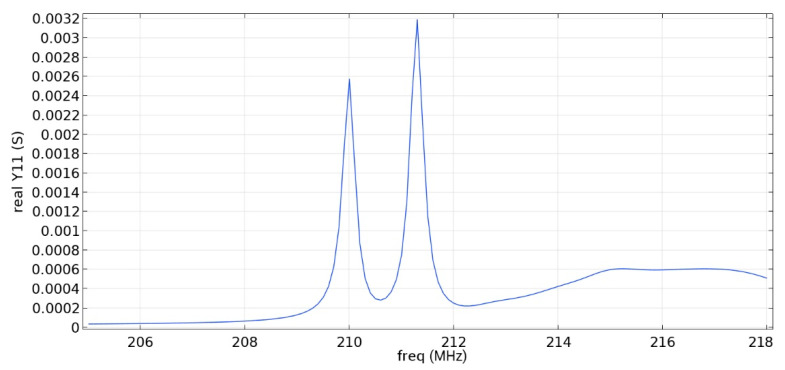
Real component of admittance for the seventh type of IDT.

**Figure 19:**
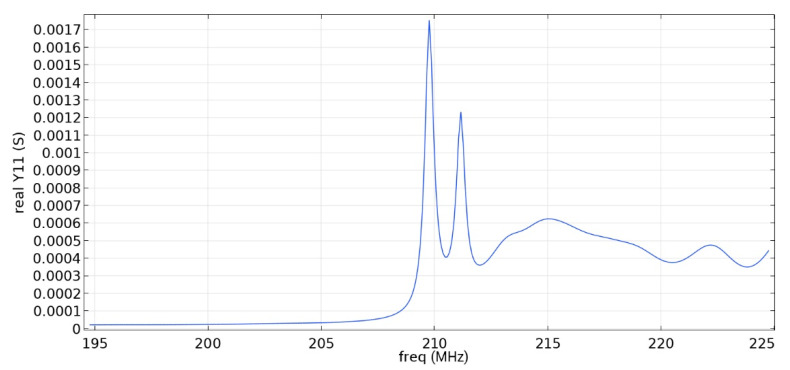
Real component of admittance for the eighth type of IDT.

**Figure 20:**
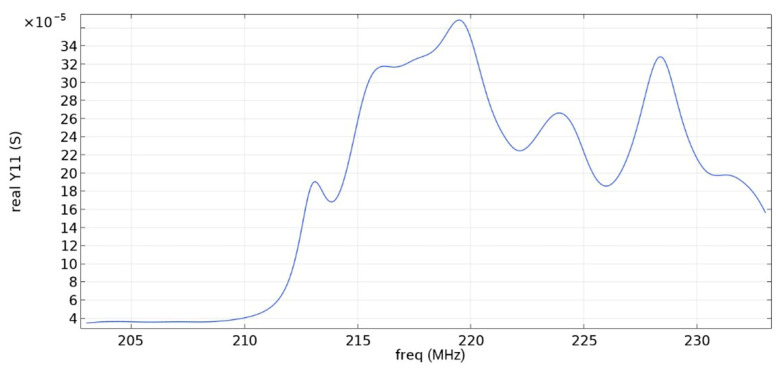
Real component of admittance for the ninth type of IDT.

**Table 1:** IDT and console parameters.

Parameter	Value
$\theta_{p}$ (angular period)	1°
R_1_ (inner radius)	1000 µm
R_2_ (outer radius)	1120 µm
h (IDT height)	0.2 µm
W (aperture)	93 µm
R0 (console radius)	1500 µm
h0 (console height)	134.4 µm

**Table 2:** Characteristics of piezoelectric material and silicone adhesive.

Parameter	YX-128°-Cut LiNbO_3_	Silicone Adhesive
Wave velocity, *v_p_* [m/s]	3961	-
Density, *ρ* [kg/m^3^]	4640	1700
Elasticmodulus, *E* [Pa]	170 × 10^9^	25 × 10^6^
Poisson’s ratio, *v*	0.25	0.48

**Table 3:** Matrix form of the tensor of elasticity of the 4th rank of the cut YX-128° of lithium niobate (GPa).

	$c_{E}{}_{m1}$	$c_{E}{}_{m2}$	$c_{E}{}_{m3}$	$c_{E}{}_{m4}$	$c_{E}{}_{m5}$	$c_{E}{}_{m6}$
$c_{E}{}_{1n}$	202.900	69.985	57.842	12.846		0
$c_{E}{}_{2n}$	69.985	193.970	90.330	9.312		0
$c_{E}{}_{3n}$	57.842	90.330	221.160	8.003		0
$c_{E}{}_{4n}$	12.846	9.312	8.003	75.323		0
$c_{E}{}_{5n}$	0	0	0	0	56.860	−5.092
$c_{E}{}_{6n}$	0	0	0	0	−5.092	77.919

**Table 4:** Coupling matrix of the YX-128°-cut of lithium niobate (S/m^2^).

	$e_{m1}$	$e_{m2}$	$e_{m3}$	$e_{m4}$	$e_{m5}$	$e_{m6}$
$e_{1n}$	0	0	0	0	4.4724	0.2788
$e_{2n}$	−1.8805	4.4467	−1.5221	0.0674	0	0
$e_{3n}$	1.7149	−2.6921	2.3136	0.6338	0	0

**Table 5:** Matrix of the relative dielectric constant of the YX-128° cut of lithium niobate.

Parameter	$\epsilon_{rS}{}_{m1}$	$\epsilon_{rS}{}_{m2}$	$\epsilon_{rS}{}_{m3}$
$\epsilon_{rS}{}_{1n}$	43.6000	0	0
$\epsilon_{rS}{}_{2n}$	0	38.1270	−7.0055
$\epsilon_{rS}{}_{3n}$	0	−7.0055	34.6330

**Table 6:** Maximum value of the first mode and bandwidth according to the simulation results.

Types of IDT	Maximum Value of the First Mode, S	Bandwidth Value, kHz
1	0.01420	169
2	0.01592	107

**Table 7:** Maximum value of the first mode and bandwidth according to the simulation results.

Types of IDT	Maximum Value of the First Mode, S	Bandwidth Value, kHz
2	0.01592	107
3	0.00906	138
4	0.00605	196
5	0.00132	310
6	0.00091	520
7	0.00320	304
8	0.00173	345
9	-	-

## Data Availability

The data presented in this study are available on request from the corresponding author.
